# Cd16^-^Cd56^bright^ NK Cells: A Protective NK Cell Subset for Progression and Prognosis in Amyotrophic Lateral Sclerosis

**DOI:** 10.14336/Ad.2024.1597

**Published:** 2026-02-23

**Authors:** Zhenxiang Gong, Li Ba, Zehui Li, Hongyan Hou, Min Zhang

**Affiliations:** ^1^department of Neurology, Tongji Hospital, Tongji Medical College, Huazhong University of Science and Technology, Wuhan 430000, China.; ^2^department of Neurology, Shanxi Bethune Hospital, Shanxi Academy of Medical Sciences, Third Hospital of Shanxi Medical University, Tongji Shanxi Hospital, Taiyuan, 030032, China.; ^3^department of Laboratory Medicine, Tongji Hospital, Tongji Medical College, Huazhong University of Science and Technology, Wuhan 430000, China.; ^4^Hubei Key Laboratory of Neural Injury and Functional Reconstruction, Huazhong University of Science and Technology, Wuhan 430000, China.

**Keywords:** amyotrophic lateral sclerosis, natural killer cell, disease progression

## Abstract

Amyotrophic lateral sclerosis (ALS) is a non-neuron-autonomous disease where peripheral immune dysregulation significantly impacts disease progression. However, the immunopathological mechanisms of natural killer (NK) cells in ALS remain largely unexplored. This study enrolled 241 ALS patients and 102 healthy controls (HC), analyzing lymphocyte subsets, including T cells, B cells, and NK cells. A sub-cohort of 81 ALS patients was followed up for one year at three-month intervals. Linear mixed and Cox proportional hazards models were used to evaluate the association between lymphocyte subsets and ALS progression and prognosis. Our results revealed significant reductions in total T cells, helper T cells (Th), and NK cells in ALS patients compared to HC (*P < 0.05*). Slow-progressing ALS patients exhibited higher counts of total T cells, Th, Cd16^-^Cd56^bright^ NK cells, and Cd16^+^Cd56^bright^ NK cells, while showing lower counts of Cd16^+^Cd56^dim^ NK cells compared to fast-progressing ALS patients (*P < 0.05*). ALS patients with lower Cd16^-^Cd56^bright^ NK cell counts experienced a faster decline in motor function than those with higher counts (*P < 0.05*). Elevated Cd16^-^Cd56^bright^ NK cell counts were associated with improved ALS prognosis (HR, 0.73; 95% CI: 0.60-0.90; *P < 0.05*). This study suggests that Cd16^-^Cd56^bright^ NK cells play a protective role in ALS progression and prognosis, offering a potential therapeutic target for ALS.

## INTROdUCTION

Amyotrophic lateral sclerosis (ALS) is a fatal neurodegenerative disease, and its pathogenesis and mechanisms of disease progression are still not fully understood [1]. Recent evidence has shown that ALS is a non-neuron-autonomous disease, in which peripheral immune abnormalities play a significant role in motor neuron degeneration [2]. during ALS pathology, peripheral immune cells infiltrate the spinal cord and motor cortex, affecting disease progression by modulating the phenotype and function of astrocytes and microglia [3, 4]. However, the roles of various lymphocyte subsets in ALS remain heterogeneous, and a more in-depth understanding of these subsets is crucial for identifying potential therapeutic targets.

The role of T cell subsets, particularly helper T cell (Th) subsets, in ALS has been extensively documented in recent years [5-7]. Regulatory T cells (Tregs), a key component of Th, primarily exert immunomodulatory effects through the secretion of suppressor cytokines [8]. Studies have shown that an increased count of Tregs in peripheral blood is associated with better prognosis in ALS [9-12]. However, the role of innate immune cells, such as natural killer (NK) cells, to ALS is less well understood. NK cells, as an essential component of the innate immune system, not only directly eliminate infected cells and pathogens but also modulate the activity of peripheral immune cells and glial cells within the central nervous system (CNS) [13]. Recent studies have demonstrated that modulating peripheral NK cells can influence neuroinflammation and the disease progression rate (dPR) in mouse models of ALS [14]. Several studies have preliminarily investigated changes in NK cell numbers and functions in ALS patients [14-19], but detailed characterization of NK cell subsets is still limited. The Revised ALS Functional Rating Scale (ALSFRS-R) is a tool specifically designed to assess motor function in ALS patients [20]. However, most previous studies have recorded only a single ALSFRS-R score at baseline, leading to potential inaccuracies in dPR calculations based on the initial assessment [21]. This raises concerns about the statistical validity of previous findings. Furthermore, there is a lack of research examining changes in NK cells in ALS patients within Chinese populations.

This study aims to investigate the effects of lymphocyte subsets on the dynamic progression of ALS in a cohort from Central China. To achieve this, we initially enrolled 241 ALS patients and 102 healthy controls (HC) to examine changes in routine lymphocyte subsets. ALS patients were then categorized into fast-progressing (FP) and slow-progressing (SP) groups according to the dPR at baseline. A comprehensive analysis of T cell, B cell, and NK cell subsets was conducted, comparing the FP and SP groups. Finally, the associations between lymphocyte subsets and disease progression and prognosis were assessed in a follow-up cohort. The findings of this study improve our understanding of the role of peripheral lymphocytes, particularly NK cell subsets, in ALS progression.

## MATERIALS ANd METHOdS

### Study Participants

This study was approved by the Ethics Committee of Tongji Hospital, Tongji Medical College, Huazhong University of Science and Technology (TJ-IRB2020 1219). All participants provided written informed consent, and the study adhered to the principles outlined in the Helsinki declaration. Participants were required to be at least 18 years old. ALS patients were diagnosed with ‘probable’ or ‘definite’ ALS according to the revised El Escorial criteria [22]. Individuals were excluded if they had a family history of neurodegenerative diseases, immunodeficiency, or autoimmune disorders, or if they were using any immunomodulatory medications. Laboratory tests, including erythrocyte sedimentation rate and ultrasensitive C-reactive protein, confirmed the absence of systemic inflammation in all participants. To minimize genetic variability, the study exclusively recruited Han Chinese individuals, who make up approximately 91% of the Chinese population. A total of 241 ALS patients were included, along with 102 HC included by the frequency matching on gender, age and body mass index, from January 2021 to december 2023. demographic and clinical data for all participants were meticulously recorded.

### Motor Function Assessment and Follow-up

The motor function of ALS patients was assessed using the ALSFRS-R, which consists of four subdomains, each containing three items. Each item is scored on a scale from 0 (no function) to 4 (normal function) [20]. The baseline disease progression rate (BdPR) was calculated using the formula: (48 - ALSFRS-R score at baseline) / disease duration (months). According to previous literature, patients with a BdPR ≥ 0.5 points/month at baseline were classified into the FP group, while those with a BdPR < 0.5 points/month were classified into the SP group [23]. Additionally, ALS patients were staged according to the King’s staging system, ranging from stage 1 (onset of symptoms) to stage 4 (gastrostomy tube and non-invasive ventilation) [24].

The main ALS cohort consisted of 241 ALS patients, with 100 patients in the SP group and 141 in the FP group. Patients were assessed using the ALSFRS-R scores at 3-month intervals for one year. The follow-up cohort included 81 ALS patients who completed at least two ALSFRS-R assessments within the first year after enrollment. Patients in the follow-up cohort were continuously monitored until an endpoint event occurred. Endpoint events were defined as death, tracheal intubation or tracheostomy, or permanent non-invasive ventilation (>7 days, >12 hours per day).

### Blood Sample Collection and Laboratory Tests

Ethylenediaminetetraacetic acid (EdTA)-anticoagulated peripheral blood samples were collected for complete blood counts. Meanwhile, peripheral blood samples anticoagulated with heparin were used for comprehensive biochemical analyses and flow cytometry assays.

#### Routine Blood Tests

Routine blood tests were conducted using the XN-9000 Sysmex (Sysmex Co., Kobe, Japan) following the manufacturer’s protocol. The tested parameters included red blood cell count, white blood cell count, platelet count, differential leukocyte counts (lymphocytes, neutrophils, eosinophils, basophils, monocytes), respective percentages, and hemoglobin concentrations.

#### Routine Biochemistry Tests

Routine biochemistry tests were performed using the Roche Cobas System according to the manufacturer’s instructions. The evaluated biochemical parameters included alanine aminotransferase, aspartate aminotransferase, total protein, albumin, globulin, total bilirubin, direct bilirubin, indirect bilirubin, alkaline phosphatase, gamma-glutamyl transferase, total cholesterol, triglyceride, high-density lipoprotein, low-density lipoprotein, lactate dehydrogenase, carbamide, creatinine, and uric acid.

#### Routine TBNK Lymphocytes

The methodologies for TBNK flow cytometry have been detailed in a prior study by our team [25]. Absolute counts and percentages of lymphocyte subsets, including T cells, B cells, NK cells, Cd4^+^ T cells, and Cd8^+^ T cells, were quantified using TruCOUNT tubes and the Bd Multitest 6-color TBNK Reagent Kit (Bd Biosciences) following the manufacturer’s instructions. Specifically, 50 µL of whole blood was incubated with a cocktail of monoclonal antibodies for 15 minutes at room temperature. Subsequently, 450 µL of FACS Lysing Solution was added to each sample, and analysis was performed using a FACSCanto flow cytometer.

#### T cell, B cell, and NK cell Subsets

Our team has previously described the methodologies employed for flow cytometry analysis of lymphocyte subsets in a published study [25]. Cytometry panels were specifically designed for T, B, and NK cells. Briefly, distinct monoclonal antibodies and reagents were added to 100 µL of whole blood for each panel (Supplementary Table 1-4). The samples were incubated at room temperature for 20 minutes, followed by red blood cell lysis using a lysing solution. The resulting pellets were resuspended in 300 µL of PBS and analyzed on a FACSCanto flow cytometer. For T cell subsets, we identified Th (Cd3^+^Cd4^+^), cytotoxic T cells (Ts, Cd3^+^ Cd8^+^), Cd28^+^ Th, Cd28^+^ Ts, HLA-dR^+^ T cells, HLA-dR^+^Th, HLA-dR^+^ Ts, Tregs (Cd3^+^Cd4^+^Cd25^+^ Cd127^low+^), Cd45RA^+^ Tregs and Cd45RA^-^ Tregs. For B cell subsets, we identified natural B cells (Cd3^-^Cd19^+^ Cd27^-^Igd^+^), switched memory B cells (Cd3^-^Cd19^+^ Cd27^+^Igd^-^), unswitched memory B cells (Cd3^-^Cd19^+^Cd27^+^Igd^+^) and plasma cells (Cd3^-^Cd19^+^ Cd27^+^Cd38^high^). For NK cell subsets, we identified Cd16^-^Cd56^bright^ NK cells, Cd16^+^Cd56^bright^ NK cells, Cd16^-^Cd56^dim^ NK cells, Cd16^+^Cd56^dim^ NK cells and Cd16^+^Cd56^-^ NK cells. The gating strategies of T cell, B cell, and NK cell subsets are illustrated in Supplementary Figure 1-4.

### Statistical Models and Analysis

Binary logistic regression models were employed to investigate the correlation between lymphocyte subsets and BdPR in the main ALS cohort. The results were presented as odds ratios (OR) with 95% confidence intervals (CI). Variance inflation factors (VIF) were calculated to evaluate multicollinearity among variables; a VIF value of less than 5 indicated no significant multicollinearity. The goodness of fit for the logistic regression model was assessed using the Hosmer-Lemeshow test. To examine the impact of lymphocyte subsets on dynamic disease progression in the follow-up cohort, linear mixed models (LMMs) were constructed. LMMs take into account the correlations between repeated measurements of ALSFRS-R within the same individual. Histograms of the residuals were plotted to verify the assumption of normal distribution. In LMMs, ALSFRS-R scores at different time points were first aggregated. Subsequently, the 81 ALS patients were stratified into two groups based on the median counts of each lymphocyte subset. Finally, the influence of lymphocyte subsets on dynamic disease progression was analyzed. The effects of lymphocyte subsets on ALS prognosis were evaluated using Cox proportional hazards models, with results expressed as hazard ratios (HR) and 95% CI. The proportional hazards assumption of the Cox model was assessed using the Schoenfeld residual test.

The normality of continuous variables was assessed using the Kolmogorov-Smirnov test. Continuous variables following a normal distribution were summarized as mean ± standard deviation (Sd) and compared using Student’s t-test. Non-normally distributed continuous variables were summarized as median (25th and 75th percentiles) and compared using the Mann-Whitney U-test. Categorical variables were compared using the Chi-square test. A p-value < 0.05 was considered statistically significant. All statistical analyses were conducted using R (version 4.2.0), while graphical representations were generated using FlowJo (version 10.8.1), GraphPad Prism (version 8.0), and the R package-ggplot2.

**Table 1:** demographic and clinical characteristics of participants.

Characteristics	Participants		P-value
	ALS (n = 241)	HC (n = 102)	
**Age (years)**	56 (49, 64)	52 (47, 62)	0.896^a^
**Male/Female**	150/91	65/37	0.795^b^
**Age at onset (years)**	55 ± 10	-	-
**duration (months)**	11 (6, 17)	-	-
**ALSFRS-R**	40 (37, 44)	-	-

Mann-Whitney U-test^a^ or Chi-Square test^b^ were used for statistical analysis. ALS, amyotrophic lateral sclerosis; HC, healthy controls; ALSFRS-R, the Revised ALS Functional Rating Scale.

## RESULTS

### Changes in Lymphocyte Subsets in ALS Patients

A total of 241 ALS patients and 102 age- and gender-matched HC were included in this study. No significant differences were observed in age and sex between ALS patients and HC (Table 1). The median age of onset and disease duration for ALS patients was 55 years and 11 months, respectively, with a median ALSFRS-R score of 40. No significant difference in geographical distribution was found between ALS patients and HC (Supplementary Fig. 5). All participants underwent flow cytometry analysis for routine TBNK lymphocytes. Compared to HC, ALS patients exhibited a significantly higher percentage of B cells, along with decreased counts of total peripheral lymphocytes, total T cells, Th cells, and NK cells (*P < 0.05*) (Table 2).

**Table 2:** Comparison of routine TBNK lymphocytes between ALS and HC.

Lymphocyte subsets	Participants		P-value
	ALS (n = 241)	HC (n = 102)	
**T (Cd3^+^Cd19^-^) (%)**	70.73 ± 8.16	70.83 ± 8.48	0.916^a^
**T (Cd3^+^Cd19^-^) (/μL)**	1189 (939, 1371)	1302 (1130, 1480)	0.003^b^
**B (Cd3^-^Cd19^+^) (%)**	13.90 (10.57, 17.17)	13.04 (8.77, 15.60)	0.018^b^
**B (Cd3^-^Cd19^+^) (/μL)**	224 (161, 316)	220 (146, 309)	0.579^b^
**Th (Cd3^+^Cd4^+^) (%)**	45.20 ± 7.27	44.56 ± 7.76	0.465^a^
**Th (Cd3^+^Cd4^+^) (/μL)**	760 ± 211	820 ± 247	0.025^a^
**Ts (Cd3^+^Cd8^+^) (%)**	22.14 ± 6.73	22.72 ± 6.06	0.454^a^
**Ts (Cd3^+^Cd8^+^) (/μL)**	361 (263, 467)	386 (315, 500)	0.062^b^
**NK (Cd3^-^Cd16^+^Cd56^+^) (%)**	12.81 (8.70, 18.28)	14.12 (9.53, 20.76)	0.071^b^
**NK (Cd3^-^Cd16^+^Cd56^+^) (/μL)**	191 (142, 312)	235 (165, 394)	0.009^b^
**TBNK (/μL)**	1652 (1330, 1959)	1835 (1559, 2067)	0.003^b^
**Th/Ts**	2.13 (1.60, 2.71)	1.95 (1.57, 2.52)	0.150^b^

Student’s t-test^a^ or Mann-Whitney U-test^b^ were used for statistical analysis. ALS, amyotrophic lateral sclerosis; HC, healthy controls; Th, helper T cells; Ts, cytotoxic T cells.

### Associations Between Lymphocyte Subsets and ALS Progression at Baseline

After examining the differences in lymphocyte subsets between ALS and HC, we further compared the SP and FP groups and assessed the correlation between lymphocyte subsets and BdPR. One hundred ALS patients were classified into the SP group based on their BdPR, while 141 patients were classified into the FP group. No significant differences were observed between the SP and FP groups with respect to age, sex, site of onset, King’s clinical stages, or geographical distribution. The median ALSFRS-R score in the SP group was 44, compared to 38 in the FP group (Supplementary Table 5 and Supplementary Fig. 6). Biochemical analysis revealed that aspartate aminotransferase and triglyceride levels were significantly lower in the SP group than in the FP group (*P<0.05*). No significant differences were observed in other biochemical parameters (Supplementary Table 6-8).

A comparison of routine blood indexes revealed that white blood cell and lymphocyte counts were significantly higher in the SP group compared to the FP group (*P < 0.05*) (Supplementary Table 9). Regarding T cell subsets, the SP group exhibited significantly increased counts of total T cells and Th cells, as well as a higher percentage of Cd28^+^ Th cells (*P < 0.05*) (Supplementary Table 10). No significant difference in B cell subsets was observed between the SP and FP groups (Supplementary Table 11). For NK cell subsets, the SP group exhibited significantly higher counts and percentages of Cd16^-^Cd56^bright^ NK cells and Cd16^+^Cd56^bright^ NK cells. In contrast, the percentage of Cd16^+^Cd56^dim^ NK cells was significantly lower (*P < 0.05*) (Supplementary Table 12). In the binary logistic regression model, the VIFs for all included variables were less than 5, suggesting no multicollinearity among the variables. The analysis indicated that a higher count of Cd16^-^Cd56^bright^ NK cells was associated with slower disease progression (OR, 0.547; 95% CI: 0.391-0.765; *P < 0.05*) (Table 3). The Hosmer-Lemeshow test showed a good model fit (X² = 4.302, df = 8, P = 0.829).

**Table 3:** Binary logistic regression analysis of lymphocyte subsets and disease progression.

Variables	OR (95% CI)	P-value
Sex	0.316 (0.058 - 1.719)	0.183
Age	0.970 (0.885 - 1.064)	0.524
BMI	1.082 (0.789 - 1.483)	0.625
T (/μL)	1.000 (0.993 - 1.007)	0.912
Th (/μL)	0.998 (0.989 - 1.008)	0.732
NK (/μL)	0.988 (0.969 - 1.007)	0.216
Cd28+ Th (%)	0.936 (0.769 - 1.139)	0.506
Cd16^-^Cd56^bright^ NK (/μL)	0.547 (0.391 - 0.765)	<0.001
Cd16^+^Cd56^bright^ NK (/μL)	1.069 (0.990 - 1.154)	0.089
Cd16^+^Cd56^dim^ NK (%)	1.008 (0.933 - 1.090)	0.831

OR, odds ratios; BMI, body mass index; Th, helper T cells.

### Associations Between Lymphocyte Subsets and ALS Progression and Prognosis

**Figure 1.**
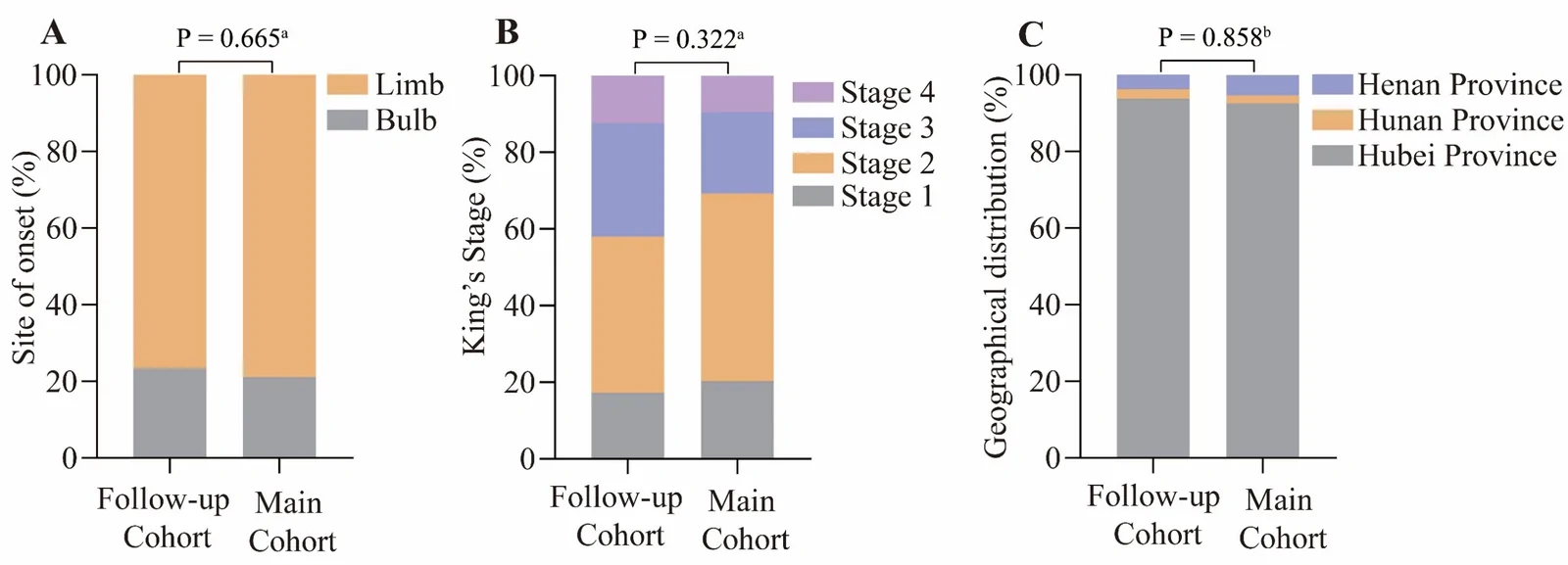
**Clinical characteristics and geographical distribution of the main cohort and the follow-up cohort**. (**A**) The proportion of onset locations in the two cohorts: limb (orange) and bulb (gray) in the follow-up cohort (n = 81) and the main cohort (n = 241). (**B**) distribution of participants according to King’s clinical stage: Stage 1 (gray), Stage 2 (orange), Stage 3 (blue), and Stage 4 (purple) in the follow-up cohort (n = 81) and the main cohort (n = 241). (**C**) The proportion of participants from Henan Province (blue), Hunan Province (orange), and Hubei Province (gray) in the follow-up cohort (n = 81) and the main cohort (n = 241). Chi-Square test^a^ or Fisher’s exact test^b^ were used for statistical analysis.

**Figure 2.**
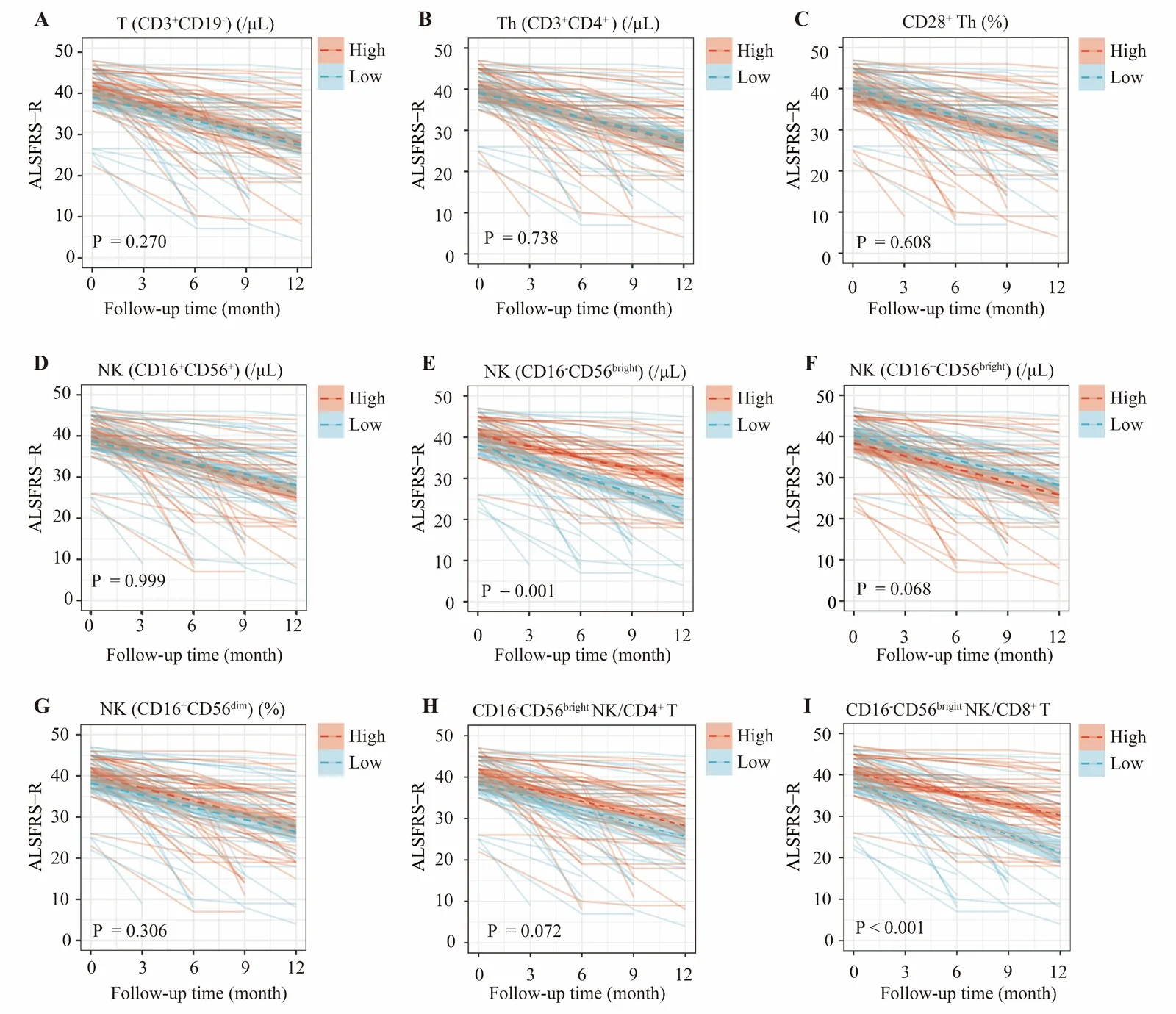
**Changes in ALSFRS-R scores over one year in relation to immune cell populations**. Patients were categorized into high (red) and low (green) groups based on the median levels of various immune cells. (**A**) T cell; (B) Th; (C) Cd28^+^ Th; (d) total NK; (E) Cd16^-^Cd56^bright^ NK cell; (F) Cd16^+^Cd56^bright^ NK cell; (G) Cd16^+^Cd56^dim^ NK cell; (H) Cd16^-^Cd56^bright^ NK cell/Cd4^+^ T cell; (I) Cd16^-^Cd56^bright^ NK cell/Cd8^+^ T cell (n = 41 in the high group, n = 40 in the low group for all comparisons). *P<0.05* indicates a statistical difference between the high and low groups in the dynamic decline of ALSFRS-R scores. ALSFRS-R, the Revised ALS Functional Rating Scale; Th, helper T cells.

**Figure 3.**
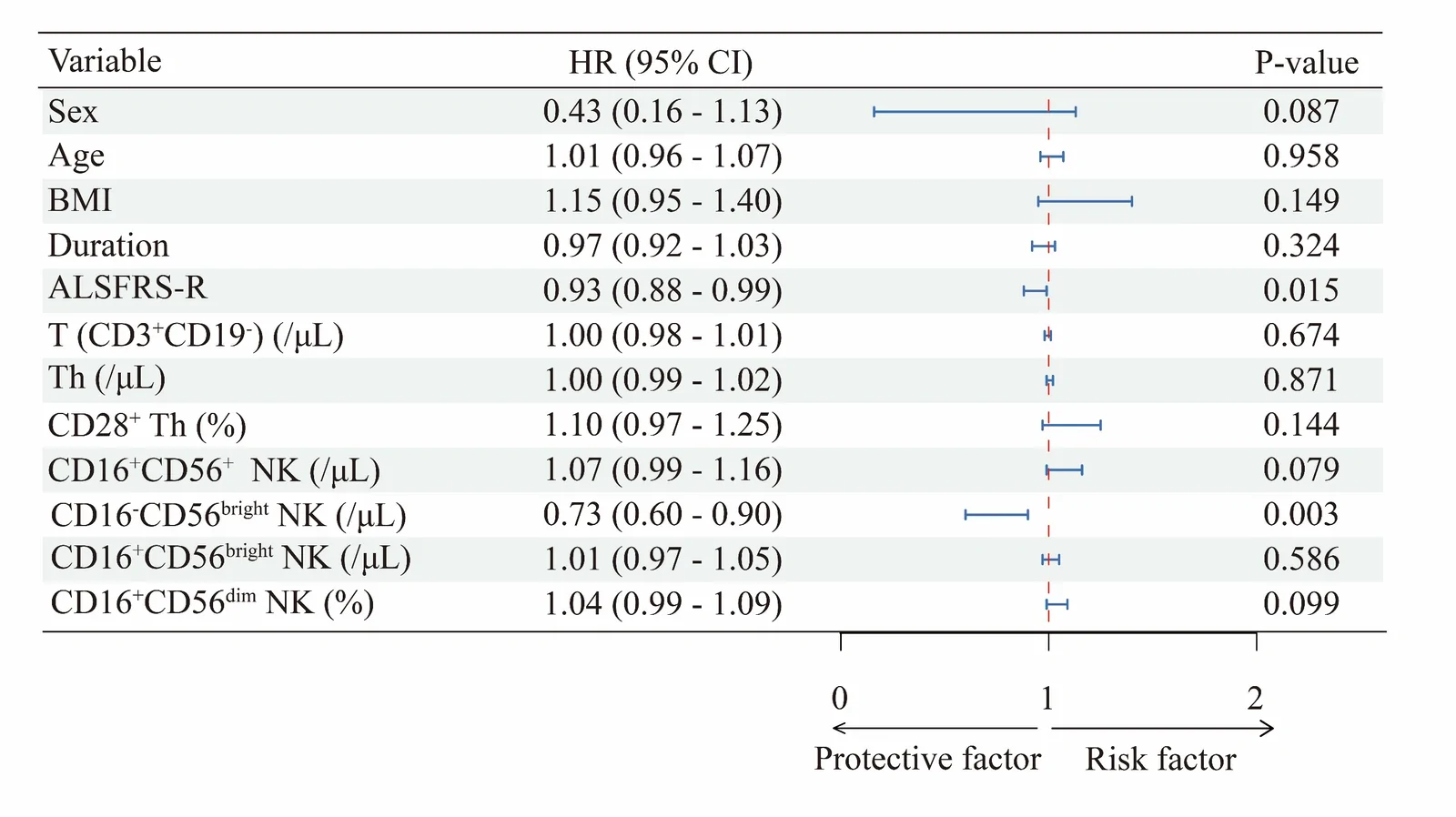
**Forest plot of Cox proportional hazard models**. The forest plot showed the effects of lymphocyte subsets on the prognosis in the follow-up cohort (n=81). HR values less than 1 indicate protective factors, and values greater than 1 indicate risk factors. *P < 0.05* indicates a statistically significant effect on the prognosis of ALS. HR, hazard ratio; BMI, body mass index; ALSFRS-R, the Revised ALS Functional Rating Scale; Th, helper T cells.

Given the heterogeneous progression of motor function in ALS patients, the BdPR may not fully capture the disease’s evolution. To address this, a subset of ALS patients was followed up after enrollment. The follow-up cohort consisted of 81 ALS patients, with demographic and clinical characteristics comparable to the primary ALS cohort (Table 4 and Fig. 1). Linear mixed models (LMMs) revealed that ALS patients with lower baseline levels of Cd16^-^Cd56^bright^ NK cells exhibited a significantly faster decline in ALSFRS-R scores within one year compared to those with higher levels (Fig. 2E). Additionally, patients with a higher ratio of Cd16^-^Cd56^bright^ NK cells to Cd8^+^ T cells demonstrated slower disease progression than those with a lower ratio (Fig. 2I). The residuals density plot indicated an approximately normal distribution (Supplementary Fig. 7). Cox proportional hazards models showed that elevated levels of Cd16-Cd56^bright^ NK cells were associated with a more favorable prognosis in ALS (HR, 0.73; 95% CI: 0.60-0.90; *P < 0.05*) (Fig. 3). The Schoenfeld residual test confirmed that the proportional hazards assumption was met (P > 0.05) (Supplementary Table 13).

**Table 4:** demographic and clinical characteristics of the main cohort and the follow-up cohort.

Characteristics	Cohort		P-value
	Follow-up cohort (n=81)	Main cohort (n=241)	
**Age (years)**	56 ± 9	56 ± 10	0.985^a^
**Male/Female (n)**	45/36	150/91	0.287^c^
**BMI (kg/m^2^)**	21.97 (19.94, 25.05)	21.97 (20.44, 24.64)	0.857^b^
**Age at onset (years)**	55 ± 9	55 ± 10	0.858^a^
**duration (months)**	10 (6, 17)	11 (6, 17)	0.948^b^
**ALSFRS-R**	41(37, 45)	40 (37, 44)	0.326^b^
**BdPR**	0.52 (0.31, 1.13)	0.64 (0.36, 1.21)	0.246^b^

Student’s t-test^a^, Mann-Whitney U-test^b^ or Chi-squared test^c^ were used for statistical analysis. BMI, body mass index; ALSFRS-R, the Revised ALS Functional Rating Scale; BdPR, baseline disease progression rate.

To further analyze the impact of Cd16^-^Cd56^bright^ NK cells on prognosis, the follow-up cohort was stratified into three groups based on NK cell counts, and a log-rank test was performed. The results showed that the group with the highest NK cell counts had a more favorable prognosis than the group with the lowest counts (P = 0.010) (Supplementary Fig. 8).

## dISCUSSION

This study investigated the differences in lymphocyte subsets between ALS patients and HC and the associations between these subsets and ALS progression. ALS patients exhibited a significant reduction in total T cells and NK cells compared to HC. In the main ALS cohort, slow-progressing patients showed significantly increased levels of total T cells, Th cells, Cd28^+^ Th cells, Cd16^-^Cd56^bright^ NK cells, and Cd16^+^Cd56^bright^ NK cells, along with decreased levels of Cd16^+^Cd56^dim^ NK cells. In the follow-up ALS cohort, increased Cd16^-^Cd56^bright^ NK cells were identified as protective factors for disease progression and prognosis. These findings suggest that, in addition to Th subsets, specific NK cell subsets also play a crucial role in ALS progression.

In the main ALS cohort, an increase in Th cells was observed in the SP group. However, this elevated Th cell count was not found to be a protective factor for disease progression in the follow-up ALS cohort. Previous studies have explored the relationship between total Th cells and ALS progression, but no consistent conclusions have been reached [11, 26-29]. This inconsistency may arise from the fact that total Th cells consist of various subsets, each of which may exert different or opposing effects on neurodegeneration [30, 31]. For example, eomesodermin, a transcription factor, plays a critical role in chronic neuroinflammation through its expression in specific Th subsets [32]. A two-center clinical study conducted in China demonstrated that elevated expression of eomesodermin in Th cells was associated with faster progression and poorer prognosis in ALS patients [33]. Foxp3 serves as a key transcriptional regulator of Tregs, and low expression of Foxp3^+^ Tregs is linked to a phenotype that compromises suppressive functions [34]. An increase in Cd4^+^Cd25^+^Foxp3^+^ Tregs was associated with slower disease progression and a more favorable prognosis [11, 35]. This study reports, for the first time, that the percentage of Cd28^+^ Th cells is elevated in slow-progressing ALS patients. Cd28 is a co-stimulatory molecule that enhances T-cell survival, proliferation, and signaling by interacting with molecules on the surface of antigen-presenting cells [36, 37]. Cd28^+^ Th cells represent an activated Th subset that plays a key role in anti-inflammatory immune responses. The human leukocyte antigen B7-Cd28 co-stimulation pathway is essential for maintaining the number and function of Tregs [38]. A decrease in Cd28^+^ Th cells has been observed in patients with Alzheimer’s disease and Parkinson’s disease, which correlates with reduced Tregs and exacerbated neuroinflammation [39, 40]. In mouse models of Parkinson’s disease, treatment with Cd28 agonists has been shown to significantly increase Tregs and mitigate early-stage degeneration of nigrostriatal dopaminergic neurons [41]. Given that elevated systemic inflammation and decreased Tregs activity are common features of neurodegenerative diseases, further investigation into the interplay between Cd28^+^ Th cells and Tregs in ALS is warranted. Additionally, future studies should aim to provide a more detailed classification of Th subsets to better understand their role in ALS pathogenesis.

In humans, NK cells are broadly classified into two principal subsets based on the expression levels of Cd16 and Cd56: Cd16^+^Cd56^dim^ NK cells and Cd16^-^Cd56^bright^ NK cells [42]. Under physiological conditions, Cd16^-^Cd56^bright^ NK cells are predominantly found in secondary lymphoid tissues, such as the lymph nodes, spleen, and tonsils, while Cd16^+^Cd56^dim^ NK cells are primarily distributed in peripheral blood. In peripheral blood, the Cd16^+^Cd56^dim^ NK subset constitutes approximately 90% of total NK cells, whereas the Cd16^-^Cd56^bright^ NK subset accounts for about 5% [43]. Compared to their cytotoxic Cd56^dim^ counterparts, Cd56^bright^ NK cells exhibit a greater capacity for trafficking into the CNS. Cd56^bright^ NK cells express high levels of migration markers, particularly C-C chemokine receptors [44, 45]. In ALS SOd1^G93A^ mice, NK cells have been shown to accumulate within the CNS in a C-C chemokine ligand-2-dependent manner [46]. In an in vitro blood-brain barrier model using human brain microvascular endothelial cells, Cd56^bright^ NK cells were found to induce the expression of intercellular adhesion molecule-1 on endothelial cells, thus facilitating their migration into the CNS [47]. Beyond their migratory capacity, Cd56^bright^ NK cells also exhibit potent immunomodulatory effects, characterized by the secretion of elevated levels of cytokines, including interferon-γ, tumor necrosis factor, and granulocyte-macrophage colony-stimulating factor, among other mediators [48]. These migratory and immunomodulatory properties of Cd56^bright^ NK cells facilitate their regulation of systemic inflammation, including neuroinflammation. Studies have shown that both peripheral and intrathecal Cd8^+^ T cells are clonally expanded in ALS patients, with this activated T cell subset exhibiting motor neuron-targeted cytotoxicity [28, 49-51]. Activated Cd8^+^ T cells upregulate class I HLA molecules, which can be recognized by Cd160 expressed on NK cells [52]. Cliona O’Farrelly et al. showed that hepatic Cd56^bright^ NK cells induce Cd160-mediated Cd8^+^ T cell death [53]. Additionally, expanded Cd56^bright^ NK cells have been shown to protect multiple sclerosis patients by inhibiting activated T cells through contact-dependent mechanisms [54]. At present, there is limited research examining the interaction between Cd56^bright^ NK cells and Cd8^+^ T cells in ALS. However, clinical data suggest that Cd56^bright^ NK cells may exert a suppressive effect on Cd8^+^ T cells, potentially slowing ALS progression. Leoni Rolfes and colleagues found that ALS patients with slow disease progression exhibited higher levels of Cd56^bright^ NK cells in their cerebrospinal fluid than those with rapid progression. Additionally, the ratio of Cd4^+^, Cd8^+^ T cells, and monocytes to Cd56^bright^ NK cells in the cerebrospinal fluid has been identified as a potential biomarker for predicting disease progression in ALS [55]. A recent study by Tatsuo Itou et al. reported that a higher ratio of peripheral Cd16^bright^Cd56^low^ NK cells to Cd16^low^Cd56^bright^ NK cells was associated with faster disease progression [56]. In our study, ALS patients with a higher ratio of peripheral Cd16^-^Cd56^bright^ NK cells to Cd8^+^ T cells demonstrated slower disease progression. However, no statistically significant differences were observed between patients with high and low ratios of Cd16^-^Cd56^bright^ NK cells to Cd4^+^ T cells. The precise immunopathological mechanisms governing the interaction between Cd16^-^Cd56^bright^ NK cells and other immune cell subsets during ALS progression warrant further research.

The strengths of the present study include the dynamic follow-up of ALS patients and the detailed phenotyping of lymphocyte subsets. However, this study has several limitations. Firstly, due to insufficient peripheral blood samples from ALS patients at different follow-up points, only the impact of baseline lymphocyte subsets on disease progression was evaluated. Additionally, lymphocyte subsets in cerebrospinal fluid samples were not assessed. Secondly, the Gold Coast criteria could have facilitated the inclusion of ALS patients at an earlier stage than the EI diagnostic criteria [57], thereby deepening our understanding of disease progression, this approach was not utilized in the current study. Thirdly, variations in climate, diet, and genetic diversity across different geographical regions may influence immune profiles. However, all participants in this study were exclusively Han Chinese individuals who had resided in central China for an extended period, which limits the generalizability of the findings. Future studies should consider including a more diverse patient population through multicenter collaborations. Moreover, elucidating the specific immunomodulatory mechanisms of Cd16^-^Cd56^bright^ NK cells in ALS will require a combination of animal experiments and clinical trials.

In conclusion, the present study demonstrates a protective role of Cd16^-^Cd56^bright^ NK cells in the progression and prognosis of ALS. Our findings provide novel insights into the peripheral immunity-related pathological mechanisms underlying ALS. Peripheral immune cells may serve as biomarkers for clinical progression and represent potential therapeutic targets in ALS.

## Supplementary Materials

The Supplementary data can be found online at: www.aginganddisease.org/EN/10.14336/Ad.2024.1597.

## Data Availability

The datasets in this study are available from the corresponding authors upon reasonable request.
