# Supplementary material for: Cd16-Cd56bright NK Cells: A Protective NK Cell Subset for Progression and Prognosis in Amyotrophic Lateral Sclerosis
Source: Aging Dis. 2025 Feb 23;17(1):405–15. doi: 10.14336/Ad.2024.1597 (PMC12727077; doi:10.14336/Ad.2024.1597)
Supplement: Supplementary file 1 — The Supplementary data can be found online at: www.aginganddisease.org/EN/10.14336/Ad.2024.1597. [file Ad-17-1-405-s.pdf]

SUPPLEMENTARY DATA

$CD16^{-}CD56^{bright}$ NK Cells: A Protective NK Cell Subset for Progression and Prognosis in Amyotrophic Lateral Sclerosis

Zhenxiang Gong, Li Ba, Zehui Li, Hongyan Hou, Min Zhang

© 2025. Gong Z et al. Published online at http://www.aginganddisease.org/EN/10.14336/AD.2024.1597

SUPPLEMENTARY DATA

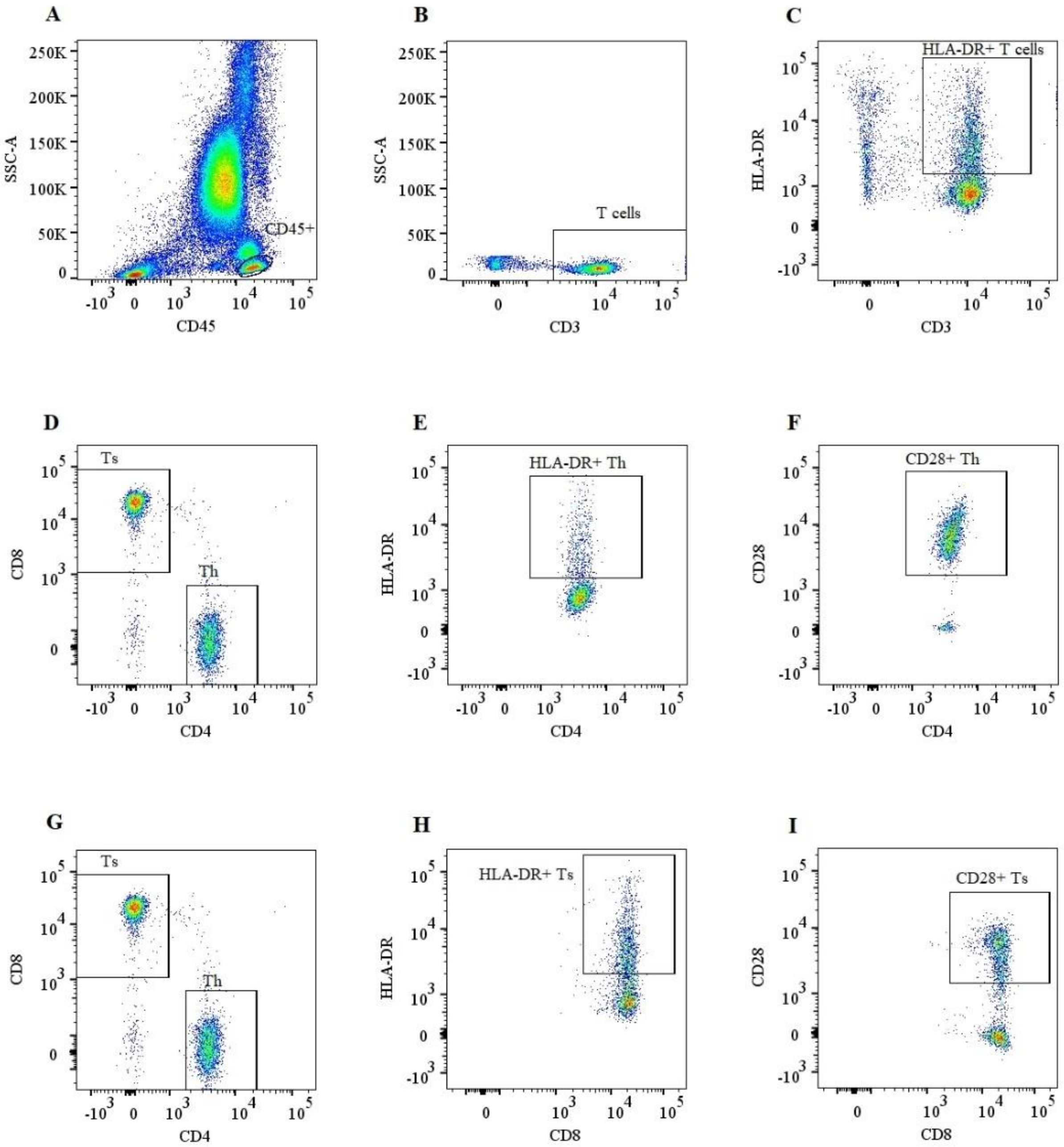

Supplementary Figure 1. Gating strategies of T cell subsets (Panel 1). SSC-A, side scatter-area; HLA-DR, human leukocyte antigen-D related; Th, helper T cells; Ts, cytotoxic T cells.

© 2025. Gong Z et al. Published online at http://www.aginganddisease.org/EN/10.14336/AD.2024.1597

SUPPLEMENTARY DATA

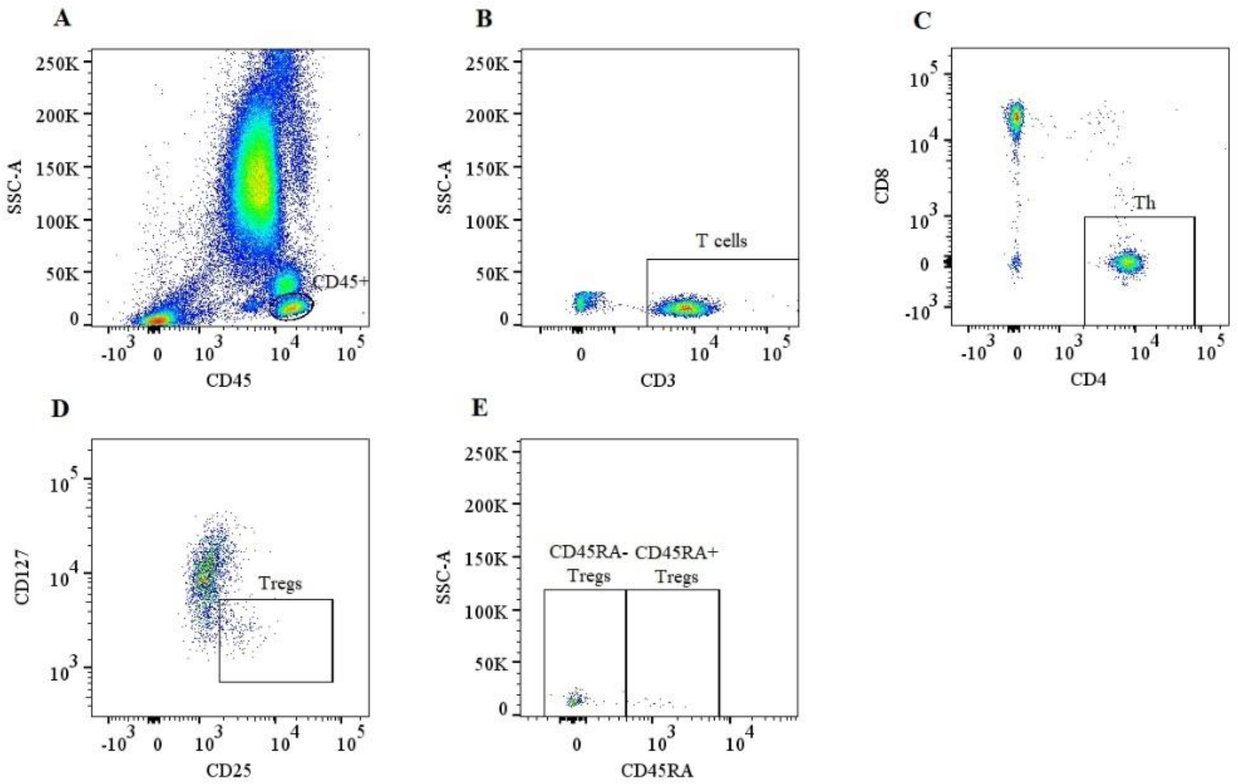

Supplementary Figure 2. Gating strategies of T cell subsets (Panel 2). SSC-A, side scatter-area; Th, helper T cells; Tregs, regulatory T cells.

© 2025. Gong Z et al. Published online at http://www.aginganddisease.org/EN/10.14336/AD.2024.1597

SUPPLEMENTARY DATA

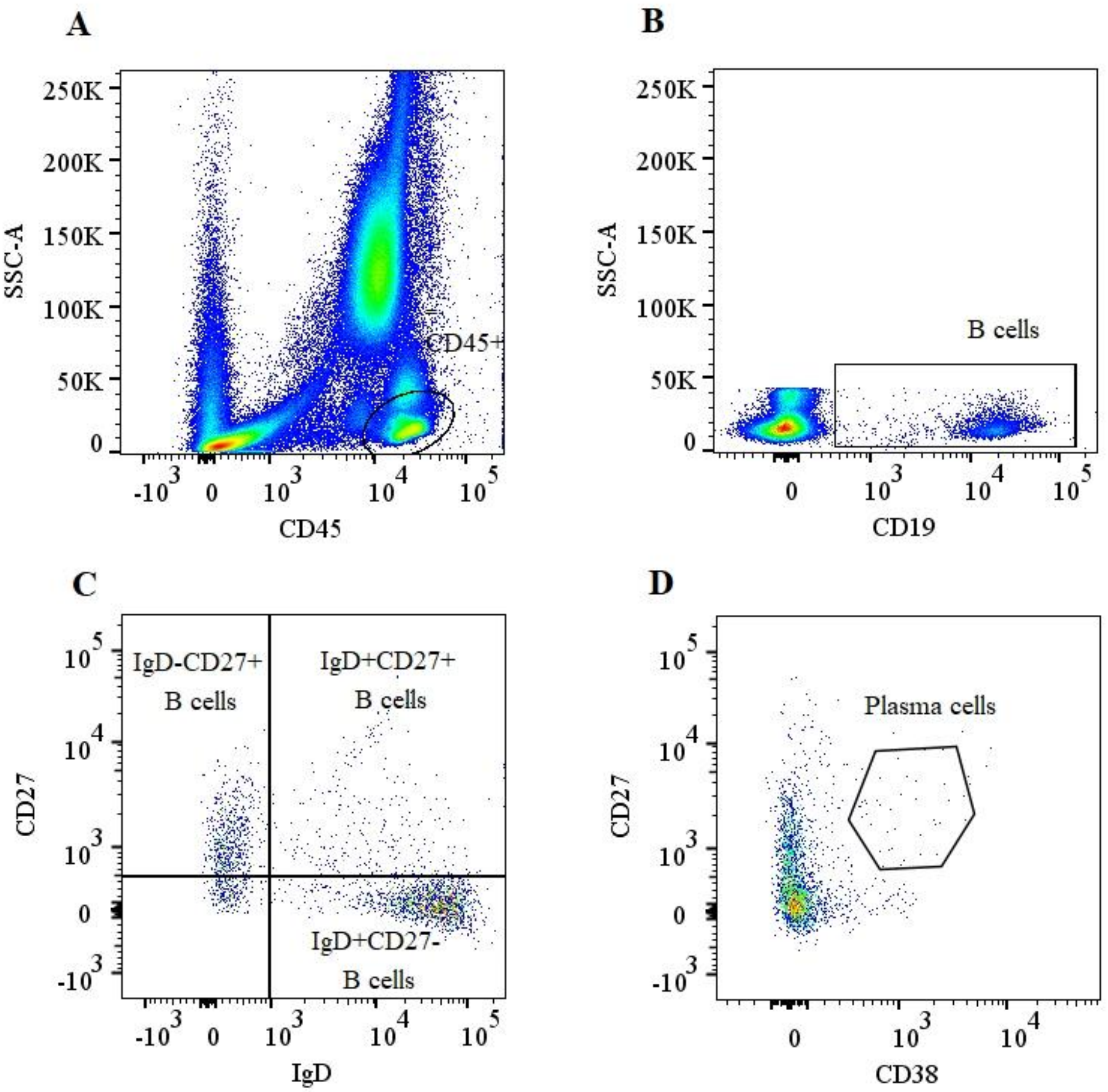

Supplementary Figure 3. Gating strategies of B cell subsets. SSC-A, side scatter-area.

© 2025. Gong Z et al. Published online at http://www.aginganddisease.org/EN/10.14336/AD.2024.1597

SUPPLEMENTARY DATA

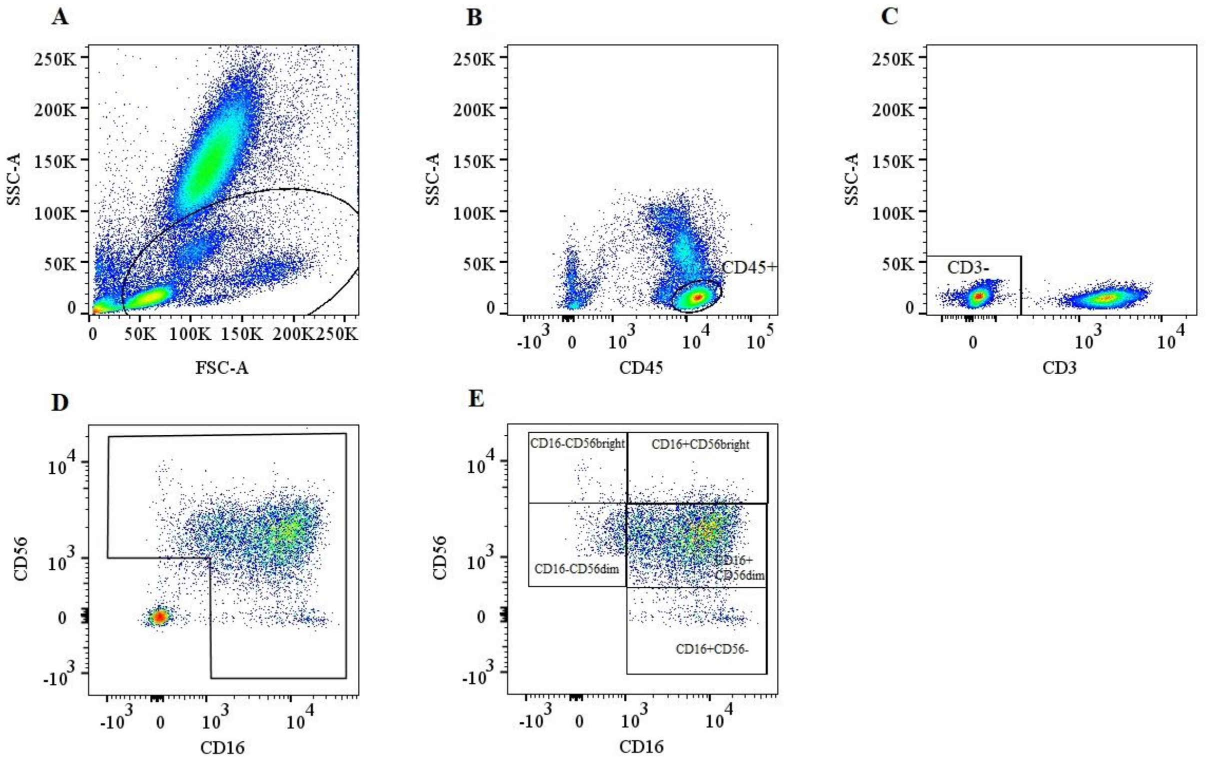

Supplementary Figure 4. Gating strategies of NK cell subsets. SSC-A, side scatter-area.

Supplementary Table 1. Antibodies for T cell subsets flow cytometry (Panel 1).

Antibodies	Catalog
anti-CD45-PerCP	BD Pharmingen, 2D1
anti-CD3- APC-H7	BD Pharmingen, SK7
anti-CD4-V500C	BD Pharmingen, RPA-T4
anti-CD8-PE/Cy7	BD Pharmingen, SK1
anti-CD28-PE	BD Pharmingen, L293
anti-HLA-DR-APC	BD Pharmingen, L243

Supplementary Table 2. Antibodies for T cell subsets flow cytometry (Panel 2).

Antibodies	Catalog
anti-CD45-PerCP	BD Pharmingen, 2D1
anti-CD3-APC-H7	BD Pharmingen, SK7
anti-CD4-V500C	BD Pharmingen, SK3
anti-CD45RA-FITC	BD Pharmingen, L48
anti-CD8-PE/Cy7	BD Pharmingen, SK1
anti-CD25-APC	BD Pharmingen, 2A3
anti-CD127-BV421	BD Pharmingen, HIL-7 R-M21

© 2025. Gong Z et al. Published online at http://www.aginganddisease.org/EN/10.14336/AD.2024.1597

Supplementary Table 3. Antibodies for B cell subsets flow cytometry.

Antibodies	Catalog
anti-CD38-FITC	BD Pharmingen, HB7
anti-CD19-PE/Cy7	BD Pharmingen, SJ25C1
anti-CD27-PerCP	BD Pharmingen, 2D1
Anti-CD45-V500C	BD Pharmingen, 2D1
anti-IgD-APC	BD Pharmingen, IA6-2

Supplementary Table 4. Antibodies for NK cell subsets flow cytometry.

Antibodies	Catalog
anti-CD45-PerCP	BD Pharmingen, 2D1
anti-CD3- FITC	BD Pharmingen, SK7
anti-CD16-PE	BD Pharmingen, B73.1
anti-CD56-PE-Cy7	BD Pharmingen, NCAM1

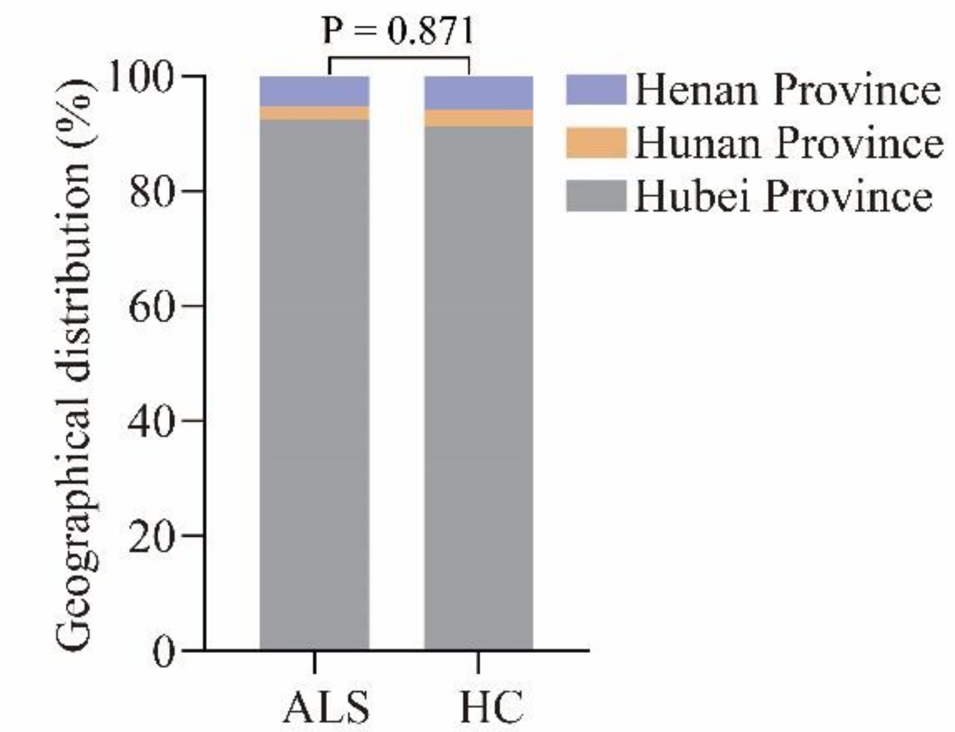

Supplementary Figure 5. Geographical distribution of ALS and HC. The proportion of participants from Henan Province (blue), Hunan Province (orange), and Hubei Province (gray) in ALS (n = 241) and HC (n = 102). Chi-Square test was used for statistical analysis. ALS, amyotrophic lateral sclerosis; HC, healthy controls.

© 2025. Gong Z et al. Published online at http://www.aginganddisease.org/EN/10.14336/AD.2024.1597

SUPPLEMENTARY DATA

Supplementary Table 5. Demographic and clinical characteristics between FP and SP.

Characteristics	ALS Patients		P-value
	**SP (n=100)**	**FP (n=141)**	
Age (years)	55 (49, 62)	58 (49, 64)	0.120[a]
Male/Female (n)	62/38	88/53	0.948[b]
BMI (kg/m^2)	22.00 (20.27, 24.93)	21.97 (20.44, 24.02)	0.628[a]
Age at onset (years)	53 (48, 60)	57 (49, 64)	0.061[a]
Duration (months)	14 (8, 24)	9 (6, 13)	**<0.001[a]**
ALSFRS-R	44 (40, 46)	38 (33, 41)	**<0.001[a]**

Mann-Whitney U-test[a] or Chi-squared test[b] were used for statistical analysis. ALS, amyotrophic lateral sclerosis; SP, slow progression group; FP, fast progression group; ALSFRS-R, the Revised ALS Functional Rating Scale.

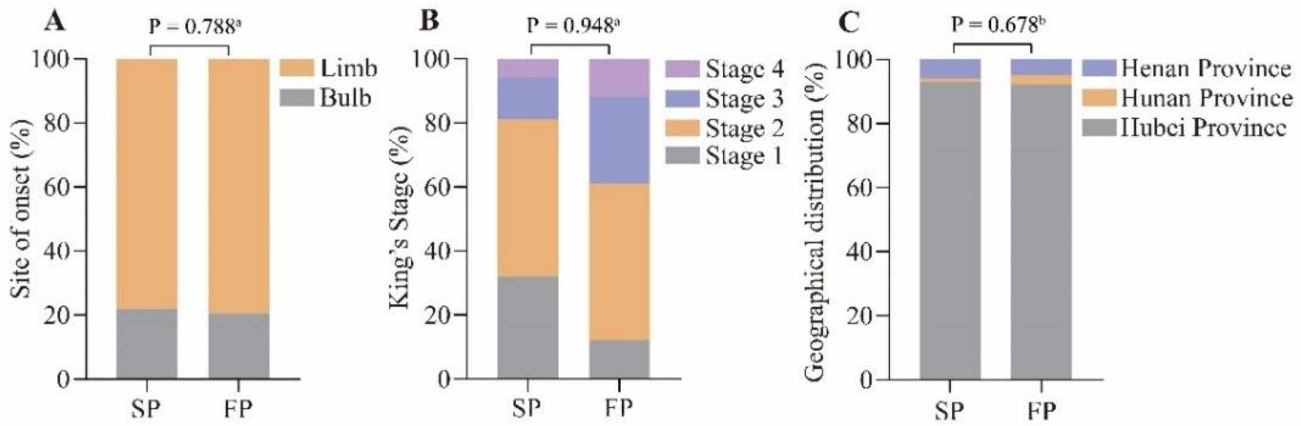

Supplementary Figure 6. Clinical characteristics and geographical distribution of the SP and FP groups. **(A)** The proportion of onset locations in the two groups: limb (orange) and bulb (gray) in the SP group (n=100) and the FP group (n=141). **(B)** Distribution of participants according to King's clinical stage: Stage 1 (gray), Stage 2 (orange), Stage 3 (blue), and Stage 4 (purple) in the SP group (n=100) and the FP group (n=141). **(C)** The proportion of participants from Henan Province (blue), Hunan Province (orange), and Hubei Province (gray) in the SP group (n=100) and the FP group (n=141). Chi-Square test[a] or Fisher's exact test[b] were used for statistical analysis. SP, slow progression group; FP, fast progression group.

Supplementary Table 6. Comparison of liver biochemical indexes between FP and SP.

Index	ALS Patients		P-value
	**SP (n=100)**	**FP (n=141)**	
ALT (U/L)	17.00 (13.00, 25.00)	20.00 (13.00, 27.50)	0.090
AST (U/L)	19.00 (16.00, 23.00)	20.00 (17.00, 26.00)	**0.015**
TP (g/L)	67.05 (65.00, 69.20)	66.00 (63.95, 69.45)	0.348
ALB (g/L)	40.60 (39.10, 42.78)	40.20 (38.60, 41.75)	0.073
GLB (g/L)	25.90 (24.00, 27.80)	26.10 (23.90, 28.50)	0.417
TBIL (umol/L)	9.85 (7.50, 12.30)	10.20 (8.15, 13.00)	0.419
DBIL (umol/L)	3.70 (3.10, 4.68)	3.80 (3.00, 4.60)	0.714
IBIL (umol/L)	6.05 (4.40, 7.80)	6.60 (5.10, 8.30)	0.238
ALP (U/L)	62.50 (53.00, 74.75)	62.00 (50.00, 74.00)	0.896
GGT (U/L)	19.00 (15.00, 28.00)	22.00 (15.50, 34.00)	0.142
LDH (U/L)	176.00 (151.50, 191.50)	179.00 (159.00, 202.00)	0.177

Mann-Whitney U-test was used for statistical analysis. ALS, amyotrophic lateral sclerosis; SP, slow progression group; FP, fast progression group; ALT, alanine aminotransferase; AST, aspartate aminotransferase; TP, total protein; ALB, albumin; GLB, globulin; TBIL, total bilirubin; DBIL, direct bilirubin; IBIL, indirect bilirubin; ALP, alkaline phosphatase; GGT, gamma-glutamyl transferase; LDH, lactate dehydrogenase.

SUPPLEMENTARY DATA

Supplementary Table 7. Comparison of kidney biochemical indexes between FP and SP.

Index	ALS Patients		P-value
	SP (n=100)	FP (n=141)	
UREA (mmol/L)	5.30 (4.34, 6.72)	5.40 (4.54, 6.28)	0.758[a]
Cr (umol/L)	63.00 (53.25, 72.75)	62.00 (49.50, 71.50)	0.400[a]
UA (umol/L)	313.65 ± 82.25	313.28 ± 95.26	0.975[b]
HCO_3 (mmol/L)	25.10 (23.05, 26.48)	24.70 (23.35, 26.30)	0.937[a]

Mann-Whitney U-test[a] or Student's t-test[b] were used for statistical analysis. ALS, amyotrophic lateral sclerosis; SP, slow progression group; FP, fast progression group; UREA, carbamide; Cr, creatinine; UA, uric acid.

Supplementary Table 8. Comparison of lipid biochemical indexes between FP and SP.

Index	ALS Patients		P-value
	SP (n=100)	FP (n=141)	
TC (mmol/L)	4.17 ± 0.84	4.31 ± 0.94	0.234[a]
TG (mmol/L)	1.21 (0.89, 1.49)	1.40 (1.01, 1.81)	**0.017[b]**
HDL (mmol/L)	1.13 (0.94, 1.28)	1.13 (0.93, 1.28)	0.539[b]
LDL (mmol/L)	2.63 ± 0.75	2.73 ± 0.80	0.320[a]

Student's t-test[a] or Mann-Whitney U-test[b] were used for statistical analysis. ALS, amyotrophic lateral sclerosis; SP, slow progression group; FP, fast progression group; TC, total cholesterol; TG, triglyceride; HDL, high-density lipoprotein; LDL, low-density lipoprotein.

Supplementary Table 9. Comparison of routine blood tests between FP and SP.

Routine blood	ALS Patients		P-value
	SP (n=100)	FP (n=141)	
RBC (10^{12}/L)	4.43 (4.12, 4.84)	4.38 (4.02, 4.70)	0.221[b]
PLT (10^9/L)	210.83 ± 55.26	199.27 ± 58.29	0.122[a]
WBC (10^9/L)	5.58 (4.57, 6.54)	5.11 (4.36, 6.09)	**0.020[b]**
LYM (%)	31.55 (28.43, 36.10)	31.90 (26.65, 37.80)	0.948[b]
LYM (10^9/L)	1.73 (1.49, 2.10)	1.67 (1.40, 1.91)	**0.021[b]**
NE (%)	58.10 (51.08, 61.78)	57.60 (51.25, 62.90)	0.906[b]
NE (10^9/L)	3.19 (2.45, 3.78)	2.93 (2.19, 3.80)	0.155[b]
NLR	1.88 (1.37, 2.21)	1.89 (1.39, 2.33)	0.555[b]
EON (%)	2.00 (1.20, 3.60)	2.50 (1.40, 3.50)	0.274[b]
EON (10^9/L)	0.11 (0.07, 0.18)	0.13 (0.07, 0.18)	0.794[b]
BAS (%)	0.40 (0.30, 0.60)	0.40 (0.25, 0.50)	0.397[b]
BAS (10^9/L)	0.02 (0.01, 0.03)	0.02 (0.01, 0.03)	0.136[b]
MON (%)	7.80 (6.90, 8.70)	7.80 (7.00, 9.30)	0.485[b]
MON (10^9/L)	0.43 (0.34, 0.50)	0.41 (0.33, 0.50)	0.161[b]
Hb (g/L)	134.00 (128.00, 147.00)	132.00 (122.00, 143.00)	0.052[b]

Student's t-test[a] or Mann-Whitney U-test[b] were used for statistical analysis. ALS, amyotrophic lateral sclerosis; SP, slow progression group; FP, fast progression group; RBC, red blood cell; PLT, platelet; WBC, white blood cell; LYM, lymphocyte; NE, neutrophil; NLR, neutrophil to lymphocyte ratio; EON, eosinophils; BAS, basophilia; MON, monocyte; Hb, hemoglobin.

© 2025. Gong Z et al. Published online at http://www.aginganddisease.org/EN/10.14336/AD.2024.1597

SUPPLEMENTARY DATA

Supplementary Table 10. Comparison of T cell subsets between FP and SP.

T cell subsets	ALS patients		P-value
	SP (n=100)	FP (n=141)	
T ($CD3^+CD19^-$) (%)	71.37 (65.79, 76.35)	70.80 (66.37, 76.25)	0.984[b]
T ($CD3^+CD19^-$) (/μL)	1279 ± 330	1128 ± 286	**<0.001**[a]
Th ($CD3^+CD4^+$) (%)	45.49 ± 6.94	45.00 ± 7.52	0.603[a]
Th ($CD3^+CD4^+$) (/μL)	788 (645, 926)	727 (581, 880)	**0.009**[b]
Ts ($CD3^+CD8^+$) (%)	21.87 ± 6.04	22.34 ± 7.19	0.599[a]
Ts ($CD3^+CD8^+$) (/μL)	361 (263, 468)	360 (261, 427)	0.394[b]
Th / Ts	2.19 (1.61, 2.67)	2.09 (1.59, 2.72)	0.588[b]
$CD28^+$ Th (%)	97.40 (95.12, 99.10)	96.39 (92.45, 98.48)	**0.029**[b]
$CD28^+$ Ts (%)	63.43 ± 14.97	61.55 ± 15.40	0.446[a]
$CD3^+HLA\text{-}DR^+$ T (/μL)	16 (12, 21)	16 (12, 21)	0.824
$HLA\text{-}DR^+$ Th (/μL)	15 ± 5	15 ± 6	0.900[a]
$HLA\text{-}DR^+$ Ts (/μL)	41 ± 14	40 ± 14	0.687[a]
Treg (/μL)	3 (3, 5)	3 (3, 4)	0.151[b]
$CD45RA^+$ Treg (/μL)	1 (1, 1)	1 (0, 1)	0.103[b]
$CD45RA^-$ Treg (/μL)	3 (2, 3)	3 (2, 3)	0.302[b]

Student's t-test[a] or Mann-Whitney U-test[b] were used for statistical analysis. ALS, amyotrophic lateral sclerosis; SP, slow progression group; FP, fast progression group; Th, helper T cells; Ts, cytotoxic T cells; Treg, regulatory T cells.

Supplementary Table 11. Comparison of B cell subsets between FP and SP.

B cell subsets	ALS patients		P-value
	SP (n=100)	FP (n=141)	
B ($CD3^-CD19^+$) (%)	13.13 (10.17, 17.21)	14.14 (10.74, 17.11)	0.456
B ($CD3^-CD19^+$) (/μL)	229 (171, 326)	222 (159, 306)	0.317
$CD19^+CD27^-IgD^+$ B (%)	67.65 (56.72, 74.65)	67.20 (58.80, 74.58)	0.693
$CD19^+CD27^+IgD^+$ B (%)	9.18 (5.67, 14.54)	9.25 (6.76, 12.46)	0.825
$CD19^+CD27^+IgD^-$ B (%)	14.96 (13.40, 21.67)	16.99 (11.08, 22.67)	0.929
$CD19^+CD27^+CD38^{high}$ B (%)	1.13 (0.48, 2.02)	1.37 (0.82, 2.47)	0.252

Mann-Whitney U-test was used for statistical analysis. ALS, amyotrophic lateral sclerosis; SP, slow progression group; FP, fast progression group.

Supplementary Table 12. Comparison of NK cell subsets between FP and SP.

NK cell subsets	ALS patients		P-value
	SP (n=100)	FP (n=141)	
NK ($CD3^-CD16^+CD56^+$) (%)	13.04 (9.40, 19.07)	12.48 (8.49, 18.12)	0.468
NK ($CD3^-CD16^+CD56^+$) (/μL)	225 (158, 331)	176 (132, 285)	**0.027**
$CD16^-CD56^{bright}$ (%)	4.49 (3.80, 5.41)	1.26 (1.00, 2.09)	**<0.001**
$CD16^-CD56^{bright}$ (/μL)	13 (6, 17)	3 (2, 5)	**<0.001**
$CD16^+CD56^{bright}$ (%)	3.27 (2.10, 5.71)	1.80 (0.73, 3.68)	**0.006**

© 2025. Gong Z et al. Published online at http://www.aginganddisease.org/EN/10.14336/AD.2024.1597

$CD16^{+}CD56^{bright}$ (/µL)	7 (5, 16)	4 (2, 7)	**0.001**
$CD16^{-}CD56^{dim}$ (%)	18.30 (14.05, 25.55)	15.45 (9.54, 22.85)	0.143
$CD16^{-}CD56^{dim}$ (/µL)	38 (27, 85)	27 (15, 73)	0.062
$CD16^{+}CD56^{dim}$ (%)	62.90 (49.50, 72.10)	72.15 (66.48, 76.00)	**0.002**
$CD16^{+}CD56^{dim}$ (/µL)	127 (102, 218)	123 (84, 225)	0.633
$CD16^{+}CD56^{-}$ (%)	4.94 (2.39, 7.47)	5.26 (2.75, 7.91)	0.637
$CD16^{+}CD56^{-}$ (/µL)	10 (5, 23)	9 (5, 14)	0.633

Mann-Whitney U-test was used for statistical analysis. ALS, amyotrophic lateral sclerosis; SP, slow progression group; FP, fast progression group.

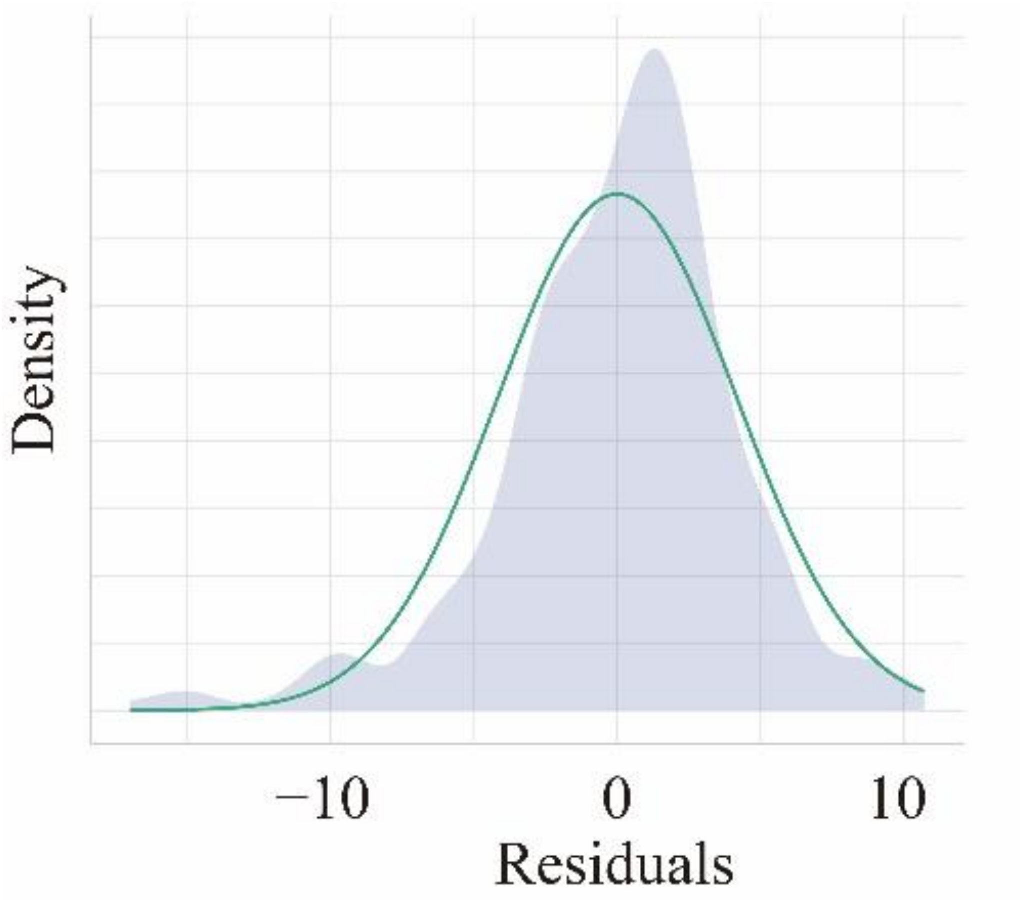

Supplementary Figure 7. Residuals density plot from linear mixed models. The residuals approximate a normal distribution, as indicated by the bell-shaped curve overlaid on the density plot.

Supplementary Table 13. Schoenfeld test of proportional hazards assumption in Cox analysis.

Variable	Chisq	P-value
Sex	0.428	0.513
Age	1.087	0.297
BMI	2.270	0.132
Duration	0.001	0.974
ALSFRS-R	0.048	0.826
T ($CD3^{+}CD19^{-}$) (/μL)	1.937	0.164
Th (/μL)	3.581	0.058
$CD28^{+}$ Th (% in Th)	2.444	0.118
NK ($CD16^{+}CD56^{+}$) (/μL)	0.062	0.080
NK ($CD16^{-}CD56^{bright}$) (/μL)	2.439	0.118
NK ($CD16^{+}CD56^{bright}$) (/μL)	0.782	0.377
NK ($CD16^{+}CD56^{dim}$) (%)	0.111	0.739
Global	18.074	0.113

Schoenfeld residuals test was used for statistical analysis. BMI, body mass index; ALSFRS-R, Revised ALS Functional Rating Scale; Th, helper T cells.

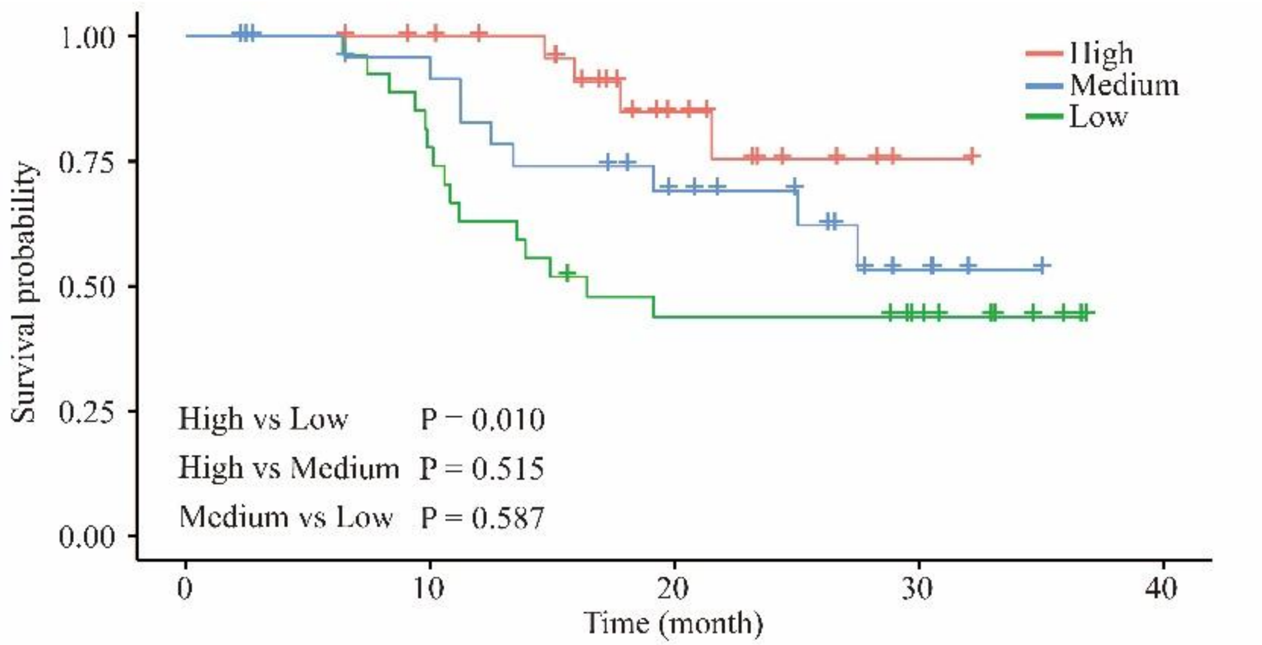

Supplementary Figure 8. Survival curves of ALS patients with different levels of $CD16^{-}CD56^{bright}$ NK cells. Kaplan-Meier survival curves show the survival probabilities of ALS patients categorized into three groups based on $CD16^{-}CD56^{bright}$ NK cell levels: high (n = 27), medium (n = 27), and low (n = 27). Log-rank test was used for statistical analysis, and the P-values were adjusted using the Bonferroni method.

© 2025. Gong Z et al. Published online at http://www.aginganddisease.org/EN/10.14336/AD.2024.1597
